# The Role of Smart Governance in Mitigating Financial Risks in the Era of Artificial Intelligence: Evidence from Iraqi Banks

**DOI:** 10.12688/f1000research.175117.1

**Published:** 2026-02-07

**Authors:** Shuaib Mohammed Sharif Abdo, Mohammed Ibrahim Mohammed Al-Jubouri, Suzan Abdul Ghani Ali, Yasir Jihad Saeed

**Affiliations:** 1 Business Administration, University of Kirkuk, Kirkuk, Kirkuk Governorate, 36001, Iraq; 2 Business Administration, University of Kirkuk, Kirkuk, Kirkuk Governorate, Iraq; 3 Business Administration, University of Kirkuk, Kirkuk, Kirkuk Governorate, Iraq; 4 Business Administration, University of Kirkuk, Kirkuk, Kirkuk Governorate, Iraq

**Keywords:** Smart governance, financial risks, Iraqi banks.

## Abstract

This paper explores how smart governance can reduce financial risks in the Iraqi banking industry by focusing on the adoption of the use of artificial intelligence (AI) technologies to increase financial stability and operational efficiency. The study uses a combination of quantitative indicators of the dimensions of smart governance (standards, policies, practices, information, technologies, and skills) and financial risk dimensions (market, credit, operational, and investment portfolio risks) based on the secondary data provided by the Central Bank of Iraq covering the years 2023 and 2024.

The results reveal a strong progression in all aspects of smart governance during the study time in the form of the rising number of licensed electronic payment providers, the rise of the percentage of current deposits, and the further use of bank accounts and electronic wallets. Also, the human resources in the banking industry have been enhanced with expertise in professional development initiatives.

In the financial risk sector, the outcomes are a decline in non-performing loans, an upward trend in the ratio of credit to deposits and an increase in total deposits and total credit facilities, hence, an indication of an improvement in the ability to handle risk. The testing of hypothesis confirms that a strong governing process, including standards, policies, practices, information management, technology adoption and development of human skills, has a positive influence on financial risk management in the AI environment.

The research suggests enhancing regulatory systems, increasing digital transformation programs, investing in human-capital growth, and introducing AI-based analytical solutions to guarantee long run sustainability and stability of the financial sector.

## Introduction

Intelligent management is a pillar towards achieving financial stability in the banking institutions. Besides paying the everyday regulatory compliance, it has a strategic management and responsive leadership integrated in it, which allows it to pass through the turbulent and changing financial environment. Of importance is the role of governance since it provides a means of balancing the interests of the shareholders, clients, and the regulatory parties through the establishment of transparency, accountability, and clearly stipulated procedural frames (Capriglione & Casalino, 2014). ^6^ Information and communication technologies have become the main driver of economic growth in most countries (developing and developed countries) because they provide the ability to achieve successes in economic growth through economic development and to bridge the gap between countries. This is likely to reduce the economic turmoil that many countries, especially developing ones, have suffered from, and these countries become able to be part of the integration of economies around the world and bring in technologies that support their efforts to achieve economic growth (Awad, Obed, & Abed, 2020). ^13^ However, banks continue to face significant obstacles, especially the need to have new forms of leadership and robust governance structures that will be able to effectively address the emerging financial risks and crisis. The human capital can work as a key to determinant here as it will help to reduce the cases of regulatory violations and strengthen the confidence of people in the banking system. (U-Din, 2021). ^22^ Regulatory structures are required but excessive dependence on them may hamper the organization flexibility. Thus, there is a new demand of new and technology-driven forms of governance that extend past the traditional designs (Bellomo & Pellerone, 2018). ^3^ Financial risk management, in its turn, is a set of strategic actions, which are undertaken to assist in minimizing the potential losses and continue running business in case of uncertainty and market variability. Artificial intelligence (AI) has transformed this area and provided predictive analytics, robotization of decisions, and data-driven insights that enable financial institutions to strive to achieve the realization of goals with less exposure to risk and fewer regulatory penalties (Sari & Indrabudiman, 2024). ^20^ The process of the introduction of artificial intelligence into the system of governance and risk management, in its turn, is transforming the global financial services sector by making the operations, strategic vision, and the customer experience more effective. It is imperative that AI-oriented systems of governance are developed in both instances of systemic risks reduction and enhancing financial resilience. It is on this basis that the present research seeks to articulate how the sophisticated systems of governance can reduce the financial vulnerabilities of the Iraqi financial institutions under the saliency of the artificial intelligence. It will do two things: firstly, to single out the key risk factors with which the banking sector of the Iraqi economy is confronted; secondly, to approximate the possibility to alleviate the dangers to a considerable extent through AI-based governance policies. Moreover, the study sheds light on the existing gap in the academic literature that fails to establish the connection between the three concepts of smart governance, artificial intelligence, and financial risk stewardship in the context of the emerging economies.

## Study methodology

**Study Problem**: The Iraqi banking institutions are trapped in the web of market, credit, and operational risks that require stringent scholarly examination.

Traditional systems of governance are considered central in the reduction of risks but they are not effective enough to counter the issues of the swift changes in technology.

With the inclusion of the concept of artificial intelligence technologies in the banking industry, a topical question arises: to what extent can the implementation of smart governance, a method combining the concept of technological penetration with the mechanisms of governance, help to increase the financial stability of the process and create a set of effective tools of oversight and transparency?

According to the above analysis, the present research can attempt to answer the following central research question: What is the role of smart governance in reducing financial risks after the implementation of artificial intelligence in Iraqi banks?

The main aim of the study is to analyze in a systematic way how intelligent governance systems will help to alleviate financial risks that arise following application of the artificial intelligence technologies. This research question is an empirical field research on the subject of the banking institutions in Iraq.

**This goal is a direct result of a number of sub-goals:**

• Dwelling upon the idea of smart governance and its aspects, and assessing its importance in enhancing the banking performance.
• Examination of nature and type of financial risks facing the Iraqi banks at the present.
• Clarifying how the aspects of smart governance and financial risks reduction in Iraqi banks relate with each other.

### Study limitations

- Spatial Limitations: Banks operating under the supervision of the Central Bank of Iraq (government and private banks).
- Temporal Limitations: 2023–2024.

### Previous studies and hypothesis development

(Bugalla et al., 2012) ^5^ “The New Model of Governance and Risk Management for Financial Institutions.”

This study proposed a new governance and risk management framework for financial institutions consisting of four components designed to enhance risk disclosure and increase stock value through improved control and management practices.

H1: Smart governance has a positive impact on reducing financial risks under artificial intelligence.

(McKinsey & Company, 2025) ^16^– This study outlined essential mechanisms to help financial institutions regulate the use of generative AI within strict governance frameworks. The analysis involved the case studies on international banking institutions and it was determined that both financial and regulatory risks involved in generative models can be significantly reduced through the application of clear-cut regulatory frameworks and the development of specialized human skills.

H2: Smart governance standards have a positive impact on reducing financial risks under artificial intelligence.

(Fritz, 2022) ^9^ – The current study aimed at formulating a systematic government structure into the assimilation of artificial intelligence models in the risk management practice of financial institutions. Based on extensive literature reviews and practical experience gained during the work of several European banks, the results prove that strong governance requires the development of clear policies regulating the risk assessment process, strict standards of testing the models, and the mechanisms aimed to maintain transparency and readability.

H3: Smart governance policies have a positive impact on reducing financial risks under artificial intelligence.

(Moridu, 2023) ^18^ – “The Role of Corporate Governance in Managing Financial Risks in Companies Listed on the West Bank Stock Exchange, Indonesia.”

This research study has considered the effects of the governance-related mechanisms, i.e., board independence, the quality of the audit committee and internal controls on the decision-making within the scope of risk management. The results indicate that a strong corporate governance greatly increases an ability of a firm to detect and assess financial risks, which contributes to their improved performance and long-term sustainability.

H4: Smart governance practices have a positive impact on reducing financial risks under artificial intelligence.

(Naguib, 2024) ^19^ – The paper examined how information technology (IT) and data governance impacted the performance of digital systems. The data were obtained by using quantitative surveys in the banking institutions and technology companies. The findings demonstrated that there is a positive relationship between advanced information governance and accuracy in financial decision-making to reduce financial risks.

H5: Smart information governance has a positive impact on reducing financial risks under artificial intelligence.

(Xinxian, 2022) ^25^ – The current paper suggested early warning model based on financial risk management using convolutional neural networks (CNNs). The proposed model based on a large amount of Chinese financial data was faster and more accurate in prediction than the traditional approaches, thus allowing the implementation of intelligent technologies to allow financial institutions to mitigate risks beforehand.

H6: Smart governance technologies have a positive impact on reducing financial risks under artificial intelligence.

(Abid et al., 2021) ^1^ – This paper has reviewed the correlation between the risk governance practices and risk taking behavior in Asian banks. It also highlighted the importance of leadership acumen in the enhancement of transparency and decision-making efficiency. The results showed that the managerial competence has a direct impact on the effectiveness of governance and financial risk management.

H7: Smart governance skills have a positive impact on reducing financial risks under artificial intelligence.

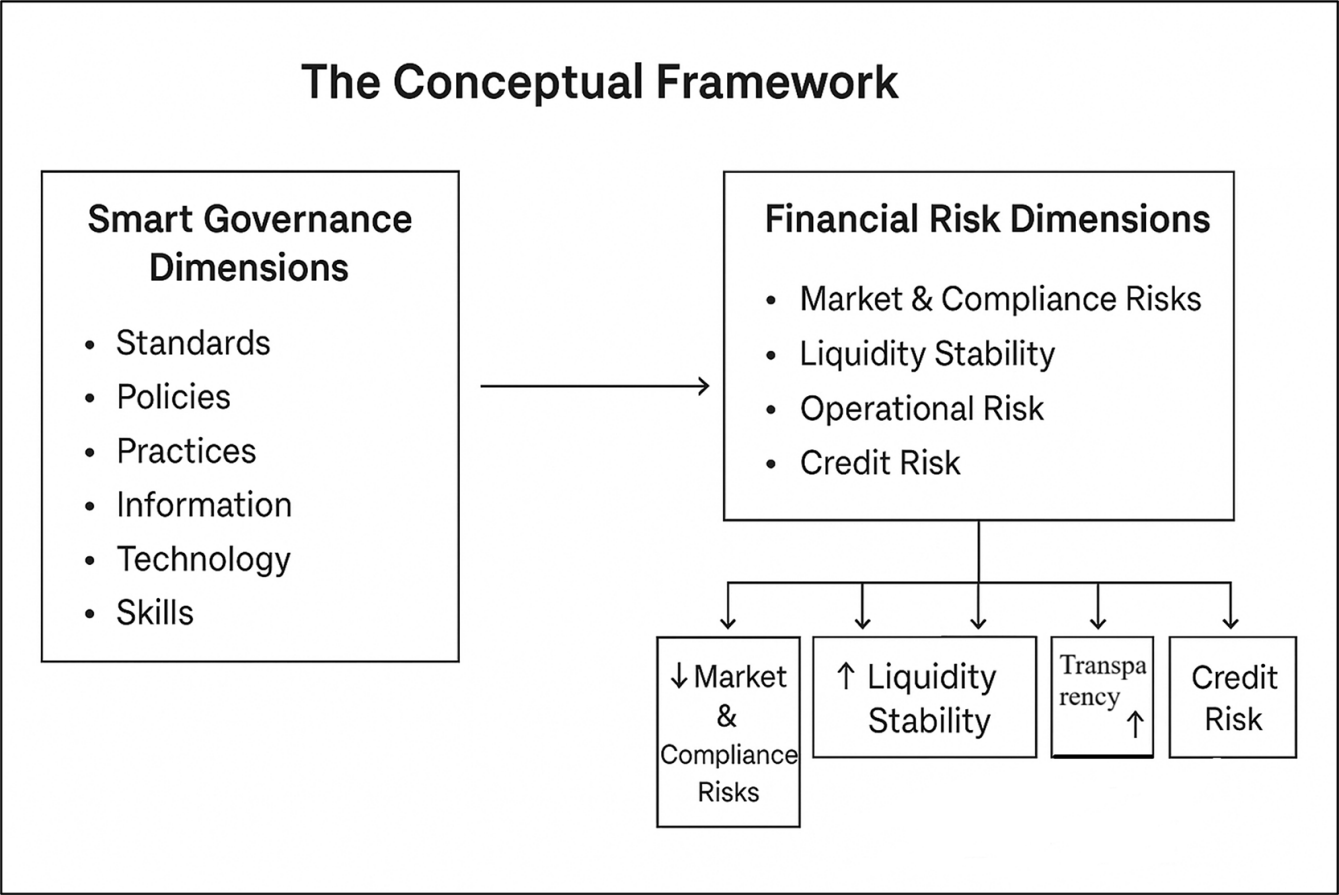


### Theoretical framework

**1. Smart Governance**

Smart governance refers to the application of digital technologies and technological advances such as artificial intelligence (AI), the Internet of Things (IoT), and big data, to improve the efficiency of the government, promote transparency and accountability, and allow citizens to be involved in the decision-making process. Therefore, this paradigm leads to the achievement of the goals of sustainable development and the quality of life increase (Jiang, 2021; Liang, Liu & Wang, 2023; Dameri, 2017). ^7, 11, 15^

Empirical studies have agreed that smart governance consists of several critical dimensions, that is, standards, policies, practices, information, technologies, as well as competencies. All dimensions play a vital and critical role in the development and execution of smart governance models. These dimensions must be synthesized in order to be able to face complex administrative challenges as well as to enhance sustainable development. The key dimensions are outlined below:

Standards:

The invention of norms and rules that introduce uniformity and quality in the public services is this dimension. Standards aid in compatibility and integration of various systems and platforms that is extremely important in good governance (Melati & Janissek, 2020; Gaulė, 2014). ^10, 17^

Policies:

Policies are road maps or blueprints that assist in guiding both decision-making and implementation of intelligent governance. They aim at promoting transparency, accountability and citizen participation according to the United Nations Sustainable Development Goals (Erza et al., 2022; Wahab et al., 2020). ^8, 24^

Practices:

The adoption of new practices aimed at improving the delivery of the public services is a part of smart governance. These are e-governance programs and participative governance system, which promote collaboration among actors and besides ease decision making processes (Gaulė, 2014; Lei, 2019). ^10, 14^

Information:

Smart governance involves the use of information, which is a constituent of the informed decision-making and strategic planning. Effective information management equips the governments to respond appropriately to the dynamics of various situations and demands by the society (Wahab et al., 2020; Bokolo & Petersen, 2019). ^4, 24^

Technologies:

Applying technologies and in particular Information and Communication Technology (ICT) would be applicable in transforming the model of governance. They may be automated to offer advanced data analytics and make smart infrastructure development in the populace and monetary institutions (Bokolo & Petersen, 2019; Azambuja et al., 2020). ^4, 2^

Skills:

This dimension also comprises the skills and expertise that the employees of the public sector are expected to possess so that they may be in a position to design, implement and maintain smart governance initiatives in a successful manner. The perpetual training and capacity building programmes are required with the view of ensuring that the staff can be able to apply the new emerging technologies (Júnior et al., 2020; Scholl & Scholl, 2014). ^12, 21^

**2. Financial Risk Management**

Financial risk management can be defined as the scientific approach of identifying, reviewing and minimizing financial risks linked to the financial operations of an organization, and its objective is to mitigate the possible losses as a result of the changes in the variables such as fluctuations in the asset prices, credit risks, and other causes of exogenous shocks which can also include the occurrence of natural disasters (Sari & Indrabudiman, 2024). ^20^

The principal categories of financial risks are outlined below:

### Market risk

Market risk refers to the changes that occur in the portfolio value of an investment due to the market fluctuations in prices or volatility. This means that any financial markets entity is automatically subjected to this type of risk. Financial institutions increasingly rely on AI technologies to enhance market risk assessment and prediction, thereby improving management efficiency (Vesna, 2021). ^23^

### Credit risk

Credit risk is the potential economic loss resulting from a counterparty’s failure to meet contractual obligations. The risks are the expected outcomes that will occur in the event of a default by the borrower or a decline in its credit quality. Machine-learning algorithms are used to predict credit events and measure the likelihood of default, therefore improving the accuracy and the effectiveness of the credit-risk management (Vesna, 2021; Sari &Indrabudiman, 2024). ^20, 23^

### Operational risk

Operational risks are often in the form of future losses due to system downtime, human mistakes, fraud, or bad internal process failures. Artificial intelligence implementation in this context is meant to support organizations in the overall operational risk management process by processing large amounts of data, automating classification efforts, and finding patterns of anomalies to eliminate risks to both internal and external stakeholders (Vesna, 2021). ^23^

### Investment portfolio management

The need to enhance investment portfolio management continues to increase and require the use of AI-driven tools. Those technologies enable the investors to manage the risks and returns more efficiently, to align the investment strategies with the institutional goals, and reduce their vulnerability to market fluctuations (Sari & Indrabudiman, 2024). ^20^

### Firstly: Study population

The target population includes the banks of Iraq which are either in the form of the public bank, the private bank or the foreign bank, under the supervision of the Central Bank of Iraq. The Central Bank is the key player that is involved in making governance policies and reducing financial risk issues that relate to the implementation of new financial technologies. The sample of the study was limited to indicators and financial records in the framework of the Central Bank of Iraq related to governance and risk management during the time frame of 2023-2024.

### Secondly: Data collection tool

The study heavily relied on secondary data that was collected through authoritative and verifiable reports published by the Central Bank of Iraq, as well as other international repositories, like the Iraq Digital Report 2024.

### Data and quantitative indicators for smart governance dimensions

Data on the main indicators that include regulatory frameworks, digital policies and regulations, e-wallet penetration, number of payment card users, and the extent of digital infrastructure (ATM and POS terminals) has been provided in Table 1. It also covers IT training programmes and banking systems. The period under analysis was 2023-2024, and data were analysed to determine progress. The given approach provides a precise and objective data collection mechanism because it will be based only on official sources of data that are credible and publicly available, which will also increase the credibility of the final results.

**Table 1.** The information related to the dimensions of smart governance in the Iraqi banking industry (2023-2024).

Dimension	Indicator details	2024 indicators	Comparison with 2023	Source
**Standards**	Number of national regulatory or guiding frameworks and initiatives related to governance, sustainable finance, and digital governance	Number of licensed electronic payment service providers: **17**	↑ from **15**	Central Bank Annual Report 2023; *Digital Iraq Report 2024*
**Policies**	Number of new digital procedures, instructions, and payment systems issued by the CBI or major regulatory amendments	Current deposits to total deposits ratio: **79.9%**	↑ from **78.5%**	Central Bank Reports 2023–2024
**Practices**	Number of e-wallets and total registered accounts (as an indicator of digital service adoption)	Number of bank accounts: **14 million**; **4,980,427** active e-wallets	↑ from **12.5 million**	Central Bank Annual Reports 2023–2024
**Information**	Number of reporting rules and transparency regulations (e.g., AI disclosure systems, updated financial statements)	Number of payment card users: **20 million**	↑ from **18 million**	Central Bank Reports 2023–2024
**Technologies**	Number of payment points (POS) and ATMs as indicators of digital infrastructure	**3,000 ATMs**; value of electronic transactions: **20 trillion IQD**	↑ from **15 trillion IQD**	CBI Press Releases
**Skills**	Number of training programs organized by the CBI in IT and banking systems	**145** training programs in 2023 (including 11 specialized IT & payment systems courses with 256 participants)	↑ in number of participants (2024)	Central Bank Annual Reports 2023–2024

**Table 2.** Data on types of financial risks in the Iraqi banking sector (2023–2024).

Dimension	Indicator	2024 indicators	Comparison with 2023	Source
**Market Risks**	Indicator: Volatility ratio direct market indicators are generally unavailable. Alternative indicator: Capital adequacy and non-performing loans ratio	Non-performing loan ratio: ↓ **7.2%**	Decrease from previous level	Central Bank Reports 2023–2024
**Credit Risks**	Indicator: Non-performing loans (NPLs) to total cash credit	Cash credit-to-deposit ratio: **57.1%**	↑ from **55%**	Central Bank Reports 2023–2024
**Operational Risks**	Indicator: Number of operational incidents and implementation of national cybersecurity controls	Total bank deposits: **127.6 trillion IQD**	↑ from **118 trillion IQD**	Central Bank Reports 2023–2024
**Investment Portfolio Management**	Indicator: Value and size of banking investment portfolios and credit granted	Credit granted: **72.7 trillion IQD**	↑ from **64.1 trillion IQD**	Central Bank Annual Reports 2023–2024

Source: Author’s own work.

The results presented in Table 1 reveal several key developments

**Table 3.** Analysis of hypothesis testing.

No.	Hypothesis	Quantitative evidence (2023–2024)	Result
**H1**	Smart governance has a positive impact on mitigating financial risks under artificial intelligence.	All dimensions (standards, policies, practices, information, technologies, and skills) improved between 2023 and 2024, reflecting a direct positive effect on risk reduction.	**Accepted**
**H2**	Smart governance standards positively influence the mitigation of financial risks under artificial intelligence.	Licensed payment service providers increased from **15** to **17**, reflecting stricter governance and lower risk of fraud or financial instability.	**Accepted**
**H3**	Smart governance policies positively affect the mitigation of financial risks under artificial intelligence.	Current deposits rose from **78.5%** to **79.9%**, enhancing liquidity and reducing the risk of sudden withdrawals.	**Accepted**
**H4**	Smart governance practices positively affect the mitigation of financial risks under artificial intelligence.	Bank accounts increased from **12.5 million** to **14 million**, with **4.98 million** e-wallets, reducing operational risks.	**Accepted**
**H5**	Smart governance information positively influences the mitigation of financial risks under artificial intelligence.	Payment card users increased from **18 million** to **20 million**, enhancing transparency and reducing credit risks.	**Accepted**
**H6**	Smart governance technologies positively affect the mitigation of financial risks under artificial intelligence.	Electronic transactions rose from **15 trillion** to **20 trillion IQD**, reducing errors and forgery risks.	**Accepted**
**H7**	Smart governance skills positively influence the mitigation of financial risks under artificial intelligence.	**145** training programs conducted in 2023 with expanded participation in 2024, reducing operational errors and improving resilience.	**Accepted**

Source: Author’s own work.

Analysis of the Results of Smart Governance Dimensions under Artificial Intelligence

a- Standards: The Central Bank of Iraq has strengthened its regulatory frameworks and national programs to strengthen governance and digital finance. It can be noted that during 2023-2024, the number of licensed electronic payment service providers increased by two, to reach seventeen, which indicates the introduction of stricter compliance requirements. This rise is an indicator of more stricter enforcement of standards according to which, only those providers are granted a license complying with the principles of governance, which promotes trust and transparency and reduces the likelihood of fraud or systemic financial instability.
b- Policies: The policies and regulations by the central banks have helped in promoting more liquidity and trust in the digital banking services. The current/total deposits ratio also improved as 78.5% in 2023 to 79.9% in 2024 which shows that the market has become more stable and there is a lower possibility that the market will experience sudden liquidity withdrawals or volatility.
c- Practices: Practical adoption of digital services such as e-wallets and electronic accounts has expanded notably. The number of bank accounts grew from 12.5 million in 2023 to 14 million in 2024, with nearly 4.98 million active e-wallets. The growth is an evidence of the growing customer dependence on digital platforms, which will increase their operational efficiency and reduce the risk factors involved in manual transactions.
d- Information: There was an increase in the payment cards base by 18 to 20 million users, and these facts allow concluding that the financial disclosure and transparency increase in the context of the digital reporting and AI-observed activity has been made. This will bring about more disclosure which will aid controlling the supervision and counter credit and compliance risks in the banking sector.
e- Technologies: Simultaneously, although the number of ATM did not decrease, but instead, it was 3,000, the amount of electronic operations was considerably expanded, growing by 15 trillion IQD to 20 trillion in 2023 to 2024. Such an increase indicates an increase in reliance on online channels, reducing mistakes in the implementation, forgery prevention, and making the financial system more predictable.
f- Skills: Human capital investment remains one of the concern areas. By 2023, 145 training programs were accomplished, 11 of which were connected with information technology and payment systems; one year later, the number of the participants of the training has risen significantly. Such a commitment to professional development improves the technical capabilities of the employees, reduces the operational risk and the institutional stability to complicated financial risks.

Analysis of Financial Risk Management under Artificial Intelligence

1. Market Risks: The market risk is the fluctuation in the interest rates and the general financial market forces which could influence the lending capacity of the banks and financial stability. Non-performing loans ratio decreased to 7.2 per cent in 2024, a decrease compared to the high rates experienced in 2023. This degradation implies that banks have increased their ability to handle market volatility and reduce the harmful impacts, and the overall stability in the sector has been enhanced, and the probability of systemic defaults has been reduced.
2. Credit Risks: Credit risk is a term used to describe the likelihood of default or a decline in credit quality by the borrowers. The percentage change in cash credit to deposits ratios showed that the ratio rose by 55 percent in 2023 to 57.1 percent in 2024, which means that the lending activities expanded proportionally without further reducing the liquidity levels. This development highlights the relevance of artificial intelligence in improving credit assessment systems and, consequently, decrease the likelihood of default and make risk-based lending more effective.
3. Operational Risks: Operational risks are a result of technical breakdown, human mistakes, or internal inefficiency. The increase in the total deposits went up to 127.6 trillion IQD in 2024 relative to 118 trillion IQD in 2023, which indicates that individuals are more persuaded about the working and cybersecurity systems of banking organizations. This enhancement implies that the introduction of advanced digital infrastructure and AI-based monitoring solutions has managed to resolve the number of internal and external incidents of operations.
4. Investment Portfolio Management: This aspect is related with the effectiveness of banking funds and investment strategies that will equalize the risk and the payoff. The credit disbursement increased to 72.7 trillion Iraqi dinars in 2024, as compared to 64.1 trillion Iraqi dinars in 2023 and shows that banks have managed to diversify the portfolios and increase investment opportunities. This expansion is coupled with the positive financial performance, improved decision-making, reinforced with the help of AI analytics, and the decrease in dependence on a few asset classes.

## Discussion of results

The current research findings demonstrate that implementing smart governance under the guidance of artificial intelligence can be described as an effective tool for mitigating financial risks in the Iraqi banking sector. Rigorous digital standards and regulatory frameworks have helped boost public confidence and increase liquidity within the sector. This aligns with the findings of the (McKinsey, 2025) report, which indicated that structured digital policies contribute to reducing emerging risks. Furthermore, IT practices have also been beneficial in eliminating operational and credit risks by enhancing transparency and oversight. Moreover, human capital and technological skills have increased employee efficiency and reduced the potential for operational errors.

Comparing 2023 and 2024, the results show a positive and objective change across all key financial indicators. Notably, the number of non-performing loans has decreased, credit has increased, and total deposits have risen. These changes indicate an improved ability of Iraqi banks to better manage and mitigate financial risks through the adoption of smart governance models. Therefore, the concept of smart governance can be viewed as a strategic tool that can be used to enhance financial stability and ensure the long-term sustainability of the banking sector in a dynamic and complex environment. Theoretically, the study contributes to filling a research gap in Arabic and international literature, by linking smart governance, artificial intelligence and financial risk management in an emerging economy environment such as Iraq.

### Recommendations

1. More stringent and open regulatory frameworks of smart governance should be set by the Central Bank of Iraq and financial institutions, and they need to be periodically reviewed to keep the pace with technological progress, as well as to reduce the risks of fraud and financial instabilities to the bare minimum.
2. The regulatory policies and procedures that would facilitate transparency, deposit protection and enhance liquidity should be increased to improve the stability of the banks to market shocks.
3. Banks ought to encourage the adoption of electronic wallet solutions and digital banking services by the customers, and thus, create operational efficiencies and reduce systemic risks caused by the use of the legacy paper-based transaction processes.
4. Clearly and transparent disclosure of financial information and digital transactions should also be developed as it increases the ability of banks to deal with credit risk and gives confidence to the investors and customers.
5. It is necessary to increase the investments in digital infrastructure, such as electronic payment networks, automated teller machines, and smart points of sale to provide operational stability and minimise the risks in those cases when some technical error or fraud will occur.
6. As a way of enhancing the competencies of employees with regards to handling complex financial risks and artificial intelligence systems, the expansion of specialized training programs in information technology and digital governance is suggested.
7. Artificial intelligence solutions should be used to process financial data and predict possible risks, which will help to preemptively and proactively make decisions that can help to prevent operational and credit risks.
8. Lastly, the enhancement of awareness campaigns in banking institutions is necessary to emphasize the strategic importance of the prudent governance and its supportive role in reducing the risk of financial risk, and therefore maintaining the adherence to the digital standards and the best practices.

## Data Availability

The dataset supporting the findings of this study is available in Zenodo at **DOI**: 10.5281/zenodo.17931357 under the CC-BY 4.0 license. **Source:** Central Bank Of Iraq \ Statistical and Research Department \ Balance Of Payments and External Trade Division. ^26^
