# Supplementary material for: The Role of Smart Governance in Mitigating Financial Risks in the Era of Artificial Intelligence: Evidence from Iraqi Banks
Source: F1000Res. 2026 May 19;15:211. Originally published 2026 Feb 7. [Version 2] doi: 10.12688/f1000research.175117.2 (PMC13213357; doi:10.12688/f1000research.175117.2)
Supplement: Peer review history

# Reviewer report: Reviewer response for version 2

**DOI:** 10.5256/f1000research.200540.r486402

**Published:** 2026-05-26

**Authors:** Istiaque Mahmud

**Affiliations:** 1 Midwestern State University, Texas, USA

The revised version has significantly improved compared with the previous submission. The authors have addressed the major concerns raised in the earlier review by reorganizing the hypotheses structure,  Overall, the revisions have strengthened the quality and analytical depth of the study, and my previous concerns have been adequately addressed.

Is the work clearly and accurately presented and does it cite the current literature?

Partly

If applicable, is the statistical analysis and its interpretation appropriate?

No

Are all the source data underlying the results available to ensure full reproducibility?

Partly

Is the study design appropriate and is the work technically sound?

No

Are the conclusions drawn adequately supported by the results?

Partly

Are sufficient details of methods and analysis provided to allow replication by others?

Partly

Reviewer Expertise:

Financial Risk Analytics, Business Analytics, Artificial Intelligence Applications in Finance, Corporate Governance, Predictive Modeling, Banking Risk Management

I confirm that I have read this submission and believe that I have an appropriate level of expertise to confirm that it is of an acceptable scientific standard.

# Reviewer report: Reviewer response for version 2

**DOI:** 10.5256/f1000research.200540.r486401

**Published:** 2026-05-26

**Authors:** Irwan Moridu

**Affiliations:** 1 Universitas Muhammadiyah Luwuk Banggai, Banggai, Indonesia

The article has been corrected according to the recommendations provided.

Is the work clearly and accurately presented and does it cite the current literature?

Partly

If applicable, is the statistical analysis and its interpretation appropriate?

No

Are all the source data underlying the results available to ensure full reproducibility?

Partly

Is the study design appropriate and is the work technically sound?

No

Are the conclusions drawn adequately supported by the results?

Partly

Are sufficient details of methods and analysis provided to allow replication by others?

Partly

Reviewer Expertise:

Financial Management, Banking, Risk Management, Financial Technology, Enterpreneurship

I confirm that I have read this submission and believe that I have an appropriate level of expertise to confirm that it is of an acceptable scientific standard.

# Reviewer report: Reviewer response for version 1

**DOI:** 10.5256/f1000research.193076.r474234

**Published:** 2026-04-23

**Authors:** Irwan Moridu

**Affiliations:** 1 Universitas Muhammadiyah Luwuk Banggai, Banggai, Indonesia

The accuracy of the presentation is only partial because several claims are stronger than the evidence provided. The study proposes multiple hypotheses, but the analysis mainly relies on descriptive comparisons between 2023 and 2024 rather than formal statistical testing, regression, or inferential analysis. 

 The manuscript cites some current literature, especially from 2020–2025, including studies on AI, governance, digital transformation, and financial risk management. Some older references are still useful for foundational theory, but the manuscript would benefit from deeper engagement with the latest empirical studies.

 The design is mainly descriptive, while the study presents several hypotheses that require stronger empirical testing. The main weakness is that the manuscript claims that smart governance has a positive impact on reducing financial risks, but it does not provide formal statistical analysis such as regression, correlation testing, econometric modeling, or hypothesis testing. The conclusions are based mostly on comparisons of indicators between 2023 and 2024, such as increases in bank accounts, e-wallets, deposits, and electronic transactions. These descriptive trends are useful, but they are not enough to prove causal or significant relationships.

 Another technical limitation is that the key concept of smart governance is not measured through a clear index or validated measurement framework. Artificial intelligence is discussed as part of the study context, but AI adoption is not directly measured using specific indicators. This makes it difficult to determine whether the observed improvement in financial risk indicators is truly caused by smart governance or AI-related governance practices.

 It also presents tables containing smart governance indicators and financial risk indicators. However, the details are not sufficient for full replication. The paper does not clearly explain how each indicator was selected, how “smart governance” was operationalized, how artificial intelligence adoption was measured, or what exact analytical procedure was used to test the hypotheses. The analysis is mainly descriptive, but the manuscript presents hypothesis results as “accepted” without showing statistical procedures, model specifications, significance levels, or robustness checks.

 The statistical analysis and interpretation are not appropriate because the manuscript presents several hypotheses as “accepted,” but it does not show formal statistical testing, regression analysis, correlation analysis, significance values, confidence intervals, or model diagnostics. The analysis is mainly based on descriptive comparisons between 2023 and 2024 indicators. Because of this, the interpretation is too strong. The study concludes that smart governance positively affects financial risk mitigation, but the evidence only shows changes in selected indicators over two years. Descriptive improvement alone cannot prove a statistically significant effect or causal relationship.

 The article states that the dataset supporting the findings is available in Zenodo  and it also identifies the Central Bank of Iraq as the main source of the statistical data. This is a positive point for transparency. However, full reproducibility is still limited because the manuscript does not clearly explain how every indicator in the tables was extracted, processed, or linked to the hypotheses. The data sources are mentioned, but the paper does not provide a detailed data processing procedure, coding framework, or statistical workflow that would allow another researcher to reproduce all results exactly.

 The conclusions are partly supported by the results. The manuscript presents descriptive evidence showing improvements in several indicators between 2023 and 2024, such as increased electronic transactions, bank accounts, deposits, credit facilities, and a decline in non-performing loans. These findings can support a general conclusion that the Iraqi banking sector showed progress in digital governance and risk related indicators. However, the conclusions are stronger than the evidence allows. The study claims that smart governance positively reduces financial risks under artificial intelligence, but the results are based mainly on descriptive comparisons rather than formal statistical testing. There is no regression, correlation analysis, causality test, or clear empirical measurement of AI adoption. Therefore, the findings can suggest a relationship, but they cannot firmly prove impact or causation.

Is the work clearly and accurately presented and does it cite the current literature?

Partly

If applicable, is the statistical analysis and its interpretation appropriate?

No

Are all the source data underlying the results available to ensure full reproducibility?

Partly

Is the study design appropriate and is the work technically sound?

No

Are the conclusions drawn adequately supported by the results?

Partly

Are sufficient details of methods and analysis provided to allow replication by others?

Partly

Reviewer Expertise:

Financial Management, Banking, Risk Management, Financial Technology, Enterpreneurship

I confirm that I have read this submission and believe that I have an appropriate level of expertise to confirm that it is of an acceptable scientific standard, however I have significant reservations, as outlined above.

# Response

**Published:** 2026-04-28

**Authors:** shuaib abdo

**Affiliations:** Business Administration, University of Kirkuk, Kirkuk, Kirkuk Governorate, Iraq

We sincerely thank the reviewer for the detailed and constructive evaluation of our manuscript. We greatly appreciate the insightful comments, which have helped us significantly improve the clarity, rigor, and methodological robustness of the study.

 ---

 Comment 1: The claims are stronger than the evidence, and the analysis is mainly descriptive.

 Response: We appreciate this important observation. In the revised manuscript, we have addressed this limitation by introducing a regression-based analytical framework (see Table 4). This addition provides formal statistical testing and allows us to examine the relationship between smart governance dimensions and financial risks beyond simple descriptive comparisons. The interpretation of results has also been carefully revised to ensure that conclusions are aligned with the empirical evidence.

 ---

 Comment 2: Lack of formal statistical testing (regression, correlation, inference).

 Response: Thank you for highlighting this issue. In response, we have incorporated regression analysis to test both the main hypothesis and sub-hypotheses. Regression coefficients (β) are now reported, showing inverse relationships between governance dimensions and financial risks. This significantly improves the empirical validity of the study and addresses the previous limitation.

 ---

 Comment 3: Smart governance is not clearly measured through a structured framework.

 Response: We have strengthened the operationalization of smart governance by clearly defining it as a multidimensional construct composed of six measurable dimensions: standards, policies, practices, information, technologies, and skills. Each dimension is now explicitly linked to quantitative indicators derived from official data (see Table 1), improving clarity, consistency, and replicability.

 ---

 Comment 4: Artificial intelligence is not directly measured.

 Response: We acknowledge this limitation. In the revised manuscript, we clarified that AI is examined within the broader context of digital governance and financial technologies. Due to data availability constraints, AI adoption is proxied through observable indicators such as digital transactions, e-payment systems, and technological infrastructure. This clarification has been explicitly added to avoid overstatement.

 ---

 Comment 5: Study design is mainly descriptive and not sufficient for hypothesis testing.

 Response: The study design has been significantly improved by integrating regression modeling alongside descriptive analysis. This allows for both trend analysis and empirical testing, strengthening the study’s technical soundness and supporting hypothesis evaluation more rigorously.

 ---

 Comment 6: Insufficient methodological detail and lack of reproducibility.

 Response: We have expanded the methodology section to provide clearer explanations of data sources, indicator selection, and analytical procedures. Additional clarification has been added regarding how variables were constructed and used in the regression model, improving transparency and reproducibility.

 ---

 Comment 7: No explanation of statistical procedures, significance, or model diagnostics.

 Response: We have added regression equations and coefficient interpretations in the revised manuscript (Table 4). While the study remains constrained by the limited time series (2023–2024), the added statistical framework improves analytical depth. We have also moderated interpretations to reflect these limitations.

 ---

 Comment 8: Data transparency is present, but processing is unclear.

 Response: We have clarified how the data were obtained from official sources and how indicators were selected and used. Additional explanation has been included to describe the linkage between raw data and study variables, improving transparency.

 ---

 Comment 9: Conclusions are stronger than the evidence allows.

 Response: We have revised the conclusions to ensure they are proportionate to the evidence provided. The language has been carefully moderated to reflect that the results indicate a strong association rather than definitive causality, given the study limitations.

 ---

 Comment 10: Need for stronger engagement with recent literature.

 Response: We have reviewed and retained recent references (2020–2025) and ensured better integration of contemporary studies within the discussion section to strengthen the theoretical and empirical grounding of the manuscript.

 ---

 Final Remark:

 We believe that these revisions have substantially improved the manuscript in terms of methodological rigor, clarity, and academic contribution. We sincerely appreciate the reviewer’s valuable feedback, which has helped us strengthen the study significantly

# Reviewer report: Reviewer response for version 1

**DOI:** 10.5256/f1000research.193076.r456738

**Published:** 2026-02-27

**Authors:** Istiaque Mahmud

**Affiliations:** 1 Midwestern State University, Texas, USA

This manuscript examines the role of smart governance and artificial intelligence in mitigating financial risks in Iraqi banks. The topic is timely and policy-relevant, and the use of official Central Bank of Iraq data enhances its practical importance.

 However, the manuscript has notable methodological limitations. Although multiple hypotheses are proposed, the study does not present formal statistical testing, regression modeling, or inferential analysis. The results rely primarily on descriptive comparisons between 2023 and 2024, which limits the ability to establish causality. Additionally, the operationalization of “smart governance” lacks a clear measurement framework, and artificial intelligence adoption is discussed conceptually rather than being empirically measured.

 The short time frame (two years) further constrains the robustness of conclusions. To strengthen the manuscript, the authors should incorporate clearer econometric methodology, define measurable governance indices, and provide proper statistical testing to support their claims.

 Overall recommendation: Major Revision.

Is the work clearly and accurately presented and does it cite the current literature?

Partly

If applicable, is the statistical analysis and its interpretation appropriate?

No

Are all the source data underlying the results available to ensure full reproducibility?

Partly

Is the study design appropriate and is the work technically sound?

No

Are the conclusions drawn adequately supported by the results?

Partly

Are sufficient details of methods and analysis provided to allow replication by others?

Partly

Reviewer Expertise:

Financial Risk Analytics, Business Analytics, Artificial Intelligence Applications in Finance, Corporate Governance, Predictive Modeling, Banking Risk Management

I confirm that I have read this submission and believe that I have an appropriate level of expertise to confirm that it is of an acceptable scientific standard, however I have significant reservations, as outlined above.

# Response

**Published:** 2026-04-28

**Authors:** shuaib abdo

**Affiliations:** Business Administration, University of Kirkuk, Kirkuk, Kirkuk Governorate, Iraq

We sincerely thank the reviewer for the constructive and insightful comments. We have carefully addressed all points raised, and the manuscript has been substantially revised to improve its methodological rigor, clarity, and empirical contribution.

 Comment 1: The manuscript lacks formal statistical testing and relies mainly on descriptive comparisons.

 Response: We appreciate this important observation. In the revised version, we have introduced a regression-based analytical framework (see Table 4) to provide formal statistical testing. The model estimates the relationship between smart governance dimensions and financial risks, with regression coefficients (β) demonstrating significant inverse relationships. This addition strengthens the study by moving beyond descriptive analysis toward causal inference.

 ---

 Comment 2: The study does not establish causality.

 Response: Thank you for this remark. To address this limitation, we incorporated regression analysis to examine the directional relationship between variables. While we acknowledge that full causality may require longer time-series or panel data, the applied model provides stronger empirical evidence of association and directional impact compared to the previous version.

 ---

 Comment 3: The operationalization of “smart governance” is unclear.

 Response: We have clarified the operationalization of smart governance by explicitly defining its six measurable dimensions (standards, policies, practices, information, technologies, and skills). Each dimension is now linked to specific quantitative indicators derived from Central Bank of Iraq data (see Table 1), improving transparency and replicability.

 ---

 Comment 4: Artificial intelligence adoption is discussed conceptually rather than empirically measured.

 Response: We acknowledge this point. In the revised manuscript, AI is more clearly integrated within the governance and technology dimensions, particularly through indicators such as digital transactions, e-payment systems, and financial technologies. We have also clarified that the study examines AI-enabled governance indirectly through observable digital transformation indicators, due to data availability constraints.

 ---

 Comment 5: The short time frame (2023–2024) limits robustness.

 Response: We agree with the reviewer. A clarification has been added in the manuscript explaining that earlier data were not sufficiently consistent or comparable. This limitation is now explicitly acknowledged, and we recommend future studies using longer time horizons.

 ---

 Comment 6: The study design and statistical analysis were not adequate.

 Response: The study design has been significantly improved through the inclusion of regression modeling and clearer variable specification. Additional explanations of the analytical approach have been added to enhance technical soundness and reproducibility.

 ---

 Comment 7: Methods and analysis are not sufficiently detailed.

 Response: We have expanded the methodology section to provide clearer explanation of data sources, variable construction, and analytical procedures. This ensures that the study can be better understood and replicated.

 ---

 Comment 8: Conclusions are not fully supported.

 Response: The conclusions have been revised to align closely with the empirical findings derived from both descriptive and regression analyses. Claims have been moderated where necessary to ensure they are fully supported by the results.

 ---

 Final Remark:

 We believe that these revisions have substantially strengthened the manuscript and addressed the reviewer’s concerns. We sincerely appreciate the reviewer’s valuable feedback, which has significantly improved the quality of this stu
